# Usability and Cultural Relevance of an mHealth App for Hispanic/Latino Individuals Living With Rheumatoid Arthritis: Protocol for a Mixed Methods Study

**DOI:** 10.2196/88401

**Published:** 2026-02-13

**Authors:** Thais F Alves, Ronnie Horner, Marie Chantel Montas, Melanie Cozad

**Affiliations:** 1 Department of Health Services Research and Administration, College of Public Health, University of Nebraska Medical Center, 42nd and Emile, Omaha, NE, 68198-4355, United States, 1 402-559-2902; 2 Department of Global Health and Population, Harvard T. H. Chan School of Public Health, Boston, MA, United States

**Keywords:** digital health intervention, mobile health, arthritis, rheumatoid, Hispanic or Latino, cultural relevance, usability

## Abstract

**Background:**

Hispanic and Latino individuals represent 14.6% of rheumatoid arthritis (RA) cases in the United States and experience significant disparities in access to rheumatologic care, disease management, and health outcomes. Mobile health (mHealth) apps are promising tools to improve patient-provider communication and self-management among populations with language and literacy barriers. However, few RA-focused digital health interventions (DHIs) have been culturally adapted for Spanish-speaking Hispanic and Latino individuals.

**Objective:**

This study aims to assess the health literacy, eHealth literacy, technology trust, and digital self-efficacy of Hispanic and Latino individuals with RA, and to evaluate the cultural relevance, usability, and patient satisfaction of the Spanish-language RunRA app. Additionally, it will explore health care providers’ perceptions of the app’s usefulness for clinical decision-making and communication with Hispanic and Latino patients.

**Methods:**

A prospective, iterative convergent mixed methods design integrated with the Framework for Reporting Adaptations and Modifications–Expanded (FRAME) will be used. We will recruit 25 Hispanic and Latino patients with RA and 7 Spanish-speaking health care professionals. Quantitative data will include standardized questionnaires (SAHL-S, eHEALS, Human-Computer Trust Scale, Digital Self-Efficacy Scale) and app analytics. Qualitative data will be collected via interviews and focus groups using the Cultural Relevance Questionnaire (CRQ), System Usability Scale (SUS), and Mobile Application Rating Scale (uMARS). Data will be analyzed using an independent intramethod strategy, with integration guided by FRAME to inform culturally relevant app modifications.

**Results:**

We anticipate enrolling 32 participants (25 patients and 7 providers). This study will be the first to evaluate the cultural relevance and usability of an mHealth app specifically designed for Spanish-speaking Hispanic and Latino individuals living with RA.

**Conclusions:**

Our long-term goal is to assess the potential for the mHealth app to act as a vehicle for the dissemination of accurate, useful, usable, and understandable health information to populations that experience health disparities and their health care providers. Findings will inform iterative refinements to RunRA and contribute to the development of culturally responsive DHIs aimed at improving communication, shared decision-making, and health outcomes in underserved populations.

## Introduction

Approximately 1.5 million people in the United States have rheumatoid arthritis (RA), with racial and ethnic minority groups, women, and elderly people experiencing a disproportionately greater burden. Hispanic and Latino individuals represent 14.6% of RA cases and paradoxically have lower risk of disease-related and treatment-related complications but they experience greater disparities in pain, fatigue, functional limitation, and risk of depression compared to non-Hispanic populations [1]. Disparities in outcomes for Hispanic and Latino individuals are associated with significant delays in obtaining initial rheumatological care related to challenges in health care access [2,3]. Challenges include low income, lower socioeconomic status, lack of health insurance, and rurality in addition to lower English language proficiency and health literacy levels [4-7]. Low ELP and health literacy for Hispanic and Latino individuals also contribute to lower levels of shared decision-making regarding treatment and symptom management and trust in providers, which may also contribute to worse outcomes [8].

Digital health interventions (DHI) are a highly promising mechanism to improve access to health care for patients with diverse language backgrounds or communication barriers, even for those in rural areas [9-11]. Appropriately designed mHealth apps represent one type of DHI with the potential to improve the patient-provider communication gap among Hispanic and Latino individuals, empowering them to obtain more patient-centric, timely, and effective care [12]. Current studies exploring the potential of culturally relevant DHIs for Hispanic and Latino individuals in the United States predominantly focus on creating behavioral change for the prevention and management of obesity, diabetes, and hypertension [10-17]. To date, only one mobile app for RA has been translated into Spanish for Hispanic and Latino patients, and limited information exists regarding the cultural relevance of RA-related DHIs for this population. To improve goal setting and shared treatment decisions among patients, our team developed ‘RunRA,’ a mHealth app designed for patients with rheumatoid arthritis. This app contains disease activity tracking, goal setting, and note-taking features that create instantaneous graphics the user can pull up on their smartphone to support shared decision-making with their provider about treatment. RunRA was recently translated into Spanish. Further refinement of this mHealth app fills an important knowledge gap by demonstrating the potential of a culturally relevant RA-related DHI to improve the experience with care of Hispanic and Latino patients with RA.

Effective use of mHealth apps as DHIs to improve share decision-making regarding treatment for RA depends on user’s engagement with the app and their willingness to use it as a frequency that provides clinical valuable information on disease activity and goal setting. As an initial step towards app refinement, this study’s objective is to assess Hispanic and Latino RA patients’ preferences for using DHIs as well as the cultural relevance, usability, and satisfaction the Spanish version of the RunRA app. It also seeks to understand providers’ perceptions on the app’s use to improve provider communication with Hispanic and Latino patients with RA.

Our specific aims are to:

1. Assess the health literacy, eHealth literacy, technology trust, and digital self-efficacy levels of Hispanic and Latino individuals living with rheumatoid arthritis;
2. Determine the cultural relevance, usability, and patient satisfaction of the mHealth app RunRA, modified for Spanish speakers, among Hispanic and Latino individuals living with rheumatoid arthritis; and
3. Assess health care providers’ perspectives on the challenges and potentialities of the RunRA app on improving patient-provider communication, and perceived usefulness of the app’s capabilities for clinical decision-making.

 The results that emerge from the qualitative and quantitative outcomes’ data analysis will allow us to track and plan the adaptations and modifications needed to improve the technology. Our findings will inform refinements to the RunRA mHealth app before its implementation and help support a future study to test its efficacy.

## Methods

### Overall Study Design

**Figure 1.**
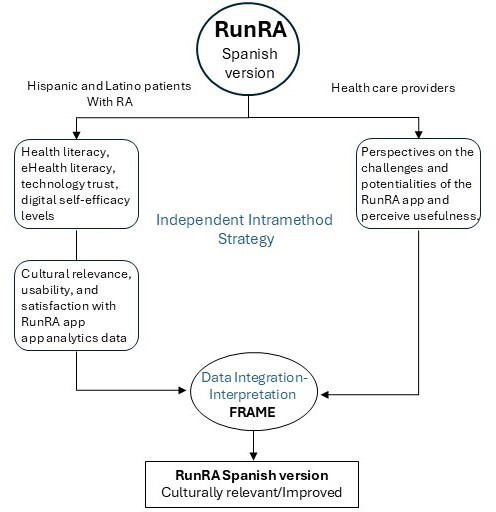
An iterative convergent mixed-method design for the Spanish RunRA evaluation. FRAME: Framework for Reporting Adaptations and Modifications-Expanded. RA: rheumatoid arthritis.

This study will apply a prospective, iterative convergent mixed methods design (ICMM) [18], integrated with the Framework for Reporting Adaptations and Modifications-Expanded (FRAME) [19]. Following ICCM, we will collect qualitative and quantitative data from Hispanic and Latino individuals living with RA and their health care providers. We will conduct an independent intra-method strategy [18] for data analyses to synthesize qualitative and quantitative data collected, and interpret those findings by applying the Integration in the Data Interpretation Dimension [18] to FRAME to determine appropriate, culturally relevant app adaptations and modifications (Figure 1). FRAME comprises eight aspects: (1) when the modification was made; (2) if it was proactive or reactive; (3) who determined that the modification should be made; (4) what is modified; (5) the level of delivery; (6) nature of content or context modifications; (7) fidelity-consistency; and (8) reasons for the modification [18]. This methodological strategy will allow us to (1) understand the app’s potential, (2) improve the technology, and (3) prepare for future evaluation of its efficacy.

#### Specific Aim 1 Design

To assess the health literacy, eHealth literacy, technology trust, and digital self-efficacy levels of Hispanic and Latino individuals living with RA, we will conduct a survey applying the Short Assessment of Health Literacy–Spanish (SAHL-S) [20], eHealth Literacy Scale (eHEALS) [21,22], the Human-computer Trust Scale [23], and the Digital Self-Efficacy Scale [24]. We will use these standardized questionnaires in their Spanish versions. This will inform us of the preferences of Hispanic or Latino patients with RA and their readiness to incorporate evidence-based health-related information delivered through technology in their health care routine.

#### Specific Aim 2 Design

To determine the cultural relevance, usability, and patient satisfaction of the mHealth app RunRA, modified for Spanish speakers, among Hispanic or Latino individuals living with RA, we will use a mixed methods approach to collect: (1) quantitative utilization data through mHealth analytics collected within the app, and (2) qualitative data through personal interviews with participants using the Cultural Relevance Questionnaire (CRQ) [25], System Usability Scale (SUS) [26], the Mobile Application Rating Scale user version (uMARS) [27], and open questions regarding app’s content.

#### Specific Aim 3 Design

For assessing health care providers’ perspectives on the challenges and potentialities of the RunRA app on improving patient-provider communication, and perceived usefulness of the app’s capabilities for clinical decision-making, we will conduct a Focus Group (FG) [28] with Spanish-speaking health professionals that assist Hispanic Latino individuals living with RA, at any level of health care (ie, nurses, community health workers, physicians). The FG script will be based on the three main categories of the CRQ: functional equivalence, conceptual equivalence, and linguistic equivalence, and on the eight aspects presented in the FRAME [19].

### Participants and Recruitment

We will recruit 25 patients for specific Aims 1 and 2, and 7 health care professionals for specific Aim 3. Patients living with RA, of Hispanic and Latino ethnicity, who are 19 years of age or older, either sex, who are Spanish-literate, and have access to a mobile device in which the RunRA app can be installed (Android or iOS operating systems) will be eligible to participate in the study. Spanish-speaking health professionals who assist Hispanic and Latino individuals living with rheumatoid arthritis in any level of health care are eligible.

To recruit participants for Aims 1 and 2, we will work with the Research, Education, Administration, and Development of Biomedical Informatics (READi) Core, which is the health informatics operations core for a large Medical Center in the Midwest. It provides cutting-edge resources to support public health research and clinical care. According to a READi query requested by our team, there are currently 2854 patients living with RA in Nebraska who opted in to participate in further research through the electronic medical record. This query included patients with a diagnosis of rheumatoid arthritis (codes M05 - M06.9) who were 19 years of age or older, either sex, any race or ethnicity, and without regard for comorbidities. For this study, we anticipate contacting up to 400 participants who are Hispanic and Latino patients with RA. This number was derived based on the national prevalence of disease among this population of 14.6%. The Center for Clinical & Translation Research Opt-In process data pull allows researchers to obtain contact information for Nebraska Medical Center patients who agreed to receive information about research opportunities when they completed their consent for treatment form. Potential participants on the READi query list will receive personalized digital fliers explaining the study and providing information to contact the researcher coordinator with their interest.

If our recruitment efforts fall behind, we will also pursue social media recruitment of participants through the University of Nebraska Medical Center for Clinical & Translation Research social media posts as a secondary strategy. Social media posts will contain information about the study and contact information for the research coordinator.

For Aim 3, we will recruit a convenience sample for the recruitment of health care professionals. When first meeting with the patients, we will ask them about their local health care providers , most often visit to help manage their condition, and based on that information, we will contact the clinics, hospitals, or health departments to invite the professionals to participate in this study.

### Data Collection of Outcomes

For Aims 1 and 2, we will collect the data in two ways – through virtual encounters and via participants’ use of the app. During our first virtual meeting, participants will answer the following questionnaires: the (SAHL-S) [20], eHEALS [21,22], the Human-computer Trust Scale [23], the Digital Self-Efficacy Scale [24]. Following completion of the survey, we will guide them on how to download and use the Spanish version of the RunRA app (Aim 1). Participants will use the app for 2 to 4 weeks [29], and our team will collect quantitative data related to mHealth app analytics (such as functions accessed, and time spent on the page) (Aim 2). We selected this time frame as it allowed sufficient time to garner appropriate user analytics following other usability studies. We will schedule a second virtual encounter with the same participants for a qualitative interview, using CRQ [25], SUS [26], the uMARS [27], and open questions regarding the app’s content.

For Aim 3, we will conduct a focus group with health care professionals who assist these patients to understand their perspectives regarding the potentialities and challenges of using the technology, and their perception of the app’s usefulness for clinical decision-making. The script will comprise three main categories of the CRQ, and the eight aspects presented at the FRAME framework (Aim 3).

All virtual encounters and the focus group will be recorded and transcribed using NVivo software for further analysis. A bilingual facilitator will conduct the interviews and focus groups, respecting the participants’ preferred language, English or Spanish. Table 1 shows the study outcomes we will explore in Aims 1, 2. and 3, including the source of data, specific measures, participants, and data collection specificity.

**Table 1.** Spanish RunRA app evaluation study outcomes.

Outcome	Source of data	Measures	Participants	Outcome data collection
Aim 1: Access the health literacy, eHealth literacy, technology trust, and digital self-efficacy levels of Hispanic/Latino individuals living with RA^a^				
Health literacy	standardized questionnaire	Short Assessment of Health Literacy–Spanish (SAHL-S)	Hispanic and Latino individuals living with RA	During the first meeting for app download
eHealth literacy	Standardized questionnaire	The eHealth Literacy Scale (eHEALS)	Hispanic and Latino individuals living with RA	During the first meeting for app download
Technology trust	Standardized questionnaire	The Human Computer Trust Scale	Hispanic and Latino individuals living with RA	During the first meeting for app download
Digital self-efficacy	Standardized questionnaire	Digital Self-Efficacy Scale	Hispanic and Latino individuals living with RA	During the first meeting for app download
App’s ability to help with RA management	Questionnaire	Survey developed by the research team	Hispanic and Latino individuals living with RA	During the first meeting for app download
Aim 2: Determine the cultural relevance, usability, and satisfaction of RunRA				
Identify the app functions used most often and to what extent	Quantitative data on mHealth app analytics	Number of times a page (ie, screen) within the app was visited, amount of time participants remained there, average views on a page per user.	Hispanic and Latino individuals living with RA	As participants use the app after initial download
Usability	Standardized questionnaire	System Usability Scale	Hispanic and Latino individuals living with RA	Within 2 to 4 weeks after app download, during the interview
Satisfaction	Standardized questionnaire	Mobile Application rating Scale user version	Hispanic and Latino individuals living with RA	Within 2 to 4 weeks after app download, during the interview
Cultural relevance, and feedback on app’s content	Qualitative, in-person interview	Semi-structured interview script based on the Cultural Relevance Questionnaire (CRQ)	Hispanic and Latino individuals living with RA	Within 2 to 4 weeks after app, during the interview
Aim 3: Identify the challenges and potentialities of the RunRA mHealth App on improving patient-provider communication				
Challenges and potentialities	Qualitative focus group	Focus Group with a script based on CRQ and FRAME	Health care professionals that assist Hispanic and Latino individuals living with RA	Following interactions with patient and app after download
Perceived usefulness of the app’s capability for clinical decision-making	Questionnaire	Survey developed by the research team	Health care professionals that assist Hispanic and Latino individuals living with RA	Following interactions with patient and app after download

a RA: rheumatoid arthritis.

### Data Analysis

For data analysis, we will apply the independent intramethod strategy [18], which implies that the qualitative data is analyzed separately from the quantitative data, followed by the integration in the data interpretation dimension [18] using the FRAME framework [19].

#### Independent Intramethod Strategy

For the qualitative data outcomes collected during the interviews and the focus group (Aims 2 and 3, respectively), two researchers will independently conduct content analysis [30] in 4 stages – decontextualization, recontextualization, categorization, and compilation – using the software NVivo. To avoid personal bias and to increase validity, the two researchers will use a codebook previously established based on the constructs of CRQ and FRAME, and will meet to garner consensus after finishing the independent coding process. The quantitative outcomes collected through surveys and apps’ use (Aim 2) will be analyzed using descriptive statistics such as means, standard deviations, and tabulations. Regarding the standardized questionnaires (Aims 1 and 2), we will follow the developers’ recommendations for scoring each instrument.

#### Integration in the Data Interpretation Dimension

Results that emerged from the qualitative and quantitative outcomes data analysis will be confronted and interpreted based on the eight aspects of FRAME to help us track and plan the adaptations and modifications needed to improve the technology (Textbox 1). In the ICMM strategy, this is the step that precedes the developer updates to the mHealth intervention and, consequently, the development of the app’s new version.

Textbox 1. **Example of integration in the data interpretation dimension [18] using FRAME [19].**

**Independent intramethod strategy results**

- Quantitative data: SAHL-S scores between 0 and 14 dimension: health literacy level
- Qualitative data: Participants’ quotes from interviews and focus group dimension: linguistic equivalence perceptions

**Merging**

Interpretation: SAHL-S (Short Assessment of Health Literacy–Spanish) score results showed that the target-population has a low literacy level, and the interviews and focus group content analysis indicates that they find app’s written information too complicated.

**Adaptation and modification tracking report**

- RunRA app improvement: Research team will revise app’s written information content and adapt grammar until achieve a lower readability level (5th grade or under)
- Adaptation and Modification FRAME (Framework for Reporting Adaptations and Modifications-Expanded) coding:

-  When: pilot planned; reactive
-  What: content level: target intervention group
-  Nature: tailoring/ tweaking/ refining
-  Goal: increase engagement and retention; improve feasibility and fit with recipients; address cultural factors
-  Reason: literacy and education level
-  Fidelity-consistency/core elements: preserved

### Ethical Considerations

Data collection for this study will commence after the approval of the University of Nebraska Medical Center Institutional Review Board and the University of Nebraska Medical Center Information Technology security team. The research coordinator will meet with interested participants virtually, explain the study to them, and then have them complete the narrative consent process. Participants will receive a copy of the narrative consent term. We will offer the participants a $50 gift card for each study data collection step, totaling $100 per patient and $50 per health care provider. Regarding data confidentiality, the data elements recorded through the mobile application will be stored on a secure, cloud-based server. The mobile application will pass the deidentifiable data to a secure cloud-based storage system that is accessed only by study leads. All information in the secure-cloud storage system will be deidentifiable as this study requires no names or personal identifiers of the participants be collected. All the qualitative interviews’ data will be stored in a deidentified manner with a numerical patient identifier. A list of the participants’ names and addresses will be kept only to provide compensation for the study following the qualitative interview. This information will only be collected if the participant wishes to be compensated for the study. This list will not be linked to any of the data collected from the participants and will be kept in a secure shared folder accessible only to the study leads.

## Results

We anticipate the enrollment of 32 participants: 25 Hispanic and Latino patients living with RA, and 7 health care professionals. We expect to recruit the first participants by April 2026 and have 100% of planned enrollment recruited by August 2026. The completion of our primary endpoint data analysis is February 2027, and the results will be reported by June 2027.

## Discussion

### Study Overview

Communication is not just about language and grammar; it is a social phenomenon that involves elements related to a person’s life context and history, relationships, how they receive and attribute meaning to information, the cultural relevance of it, literacy levels, and language proficiency. In Latin America, there are approximately ten different Spanish dialects [31], and Hispanic and Latino individuals living in the United States come from these various backgrounds, making this population culturally diverse. Therefore, while developing a DHI for Hispanic and Latino individuals, language translation is essential, but much more is needed to ensure usability, satisfaction, and adoption by these patients. Our long-term goal is to assess the potential for the mHealth app to act as a vehicle for the dissemination of accurate, useful, usable, and understandable health information to populations that experience health disparities and their health care providers. This study will be the first to determine the cultural relevance, usability, and satisfaction of a DHI designed to improve patient-provider communication for the Hispanic and Latino population living with RA. Our team’s methodological approach is novel because it integrates an ICMM [20] study design with an implementation science framework—FRAME (Figure 1).

### Potential Challenges and Mitigating Strategies

The READi is a health informatics operations core that provides cutting-edge resources to support public health research and clinical care, and one of our strengths for this study recruitment is their ability to directly contact patients who want to participate in research. For our secondary strategy, literature shows that social media recruitment is a feasible and low-cost option when working with rural communities [32,33]; however, recruiting minority groups, such as Hispanic and Latino individuals, has other challenges, including communication and language, fear, and mistrust [34]. To help mitigate these barriers, all patients’ recruitment materials will be available in Spanish, adequate for lower readability levels, and will be revised by bilingual professionals. Also, we understand that sometimes the health care workload can be challenging and impose some difficulties in reuniting all participants simultaneously for a collective discussion. So, if we cannot schedule an FG with the health care professionals, we will conduct personal interviews to address the same content.

### Methodological Strengths

We will use the ICMM steps to collect and analyze the data and then apply the Framework for Reporting Adaptations and Modifications-Expanded (FRAME) [19] for integration. Previously, FRAME has been used by researchers in public health program evaluation. It helps researchers plan for intervention scale-up through the understanding of participants’ context and improve delivery and effectiveness by tracking modifications and adaptations. To our knowledge, this is the first study to blend these approaches (ie, ICCM and FRAME) for DHI (mobile app) evaluation. The successful use of these methods has the potential to be applied more broadly for all conditions.

## Supplementary material

10.2196/88401 Peer Review Report 1 Peer review report by the Innovation Fund Committee, College of Public Health, University of Nebraska Medical Center.

## Abbreviations

CRQ: Cultural Relevance Questionnaire
DHI: digital health interventions
eHEALS: eHealth Literacy Scale
FG: Focus Group
FRAME: Framework for Reporting Adaptations and Modifications-Expanded
ICCM: Iterative Convergent mixed-method design
RA: rheumatoid arthritis
READi: Research, Education, Administration, and Development of Biomedical Informatics
SAHL-S: Short Assessment of Health Literacy–Spanish
SUS: System Usability Scale
uMARS: Mobile Application rating Scale user version
