# Supplementary material for: Usability and Cultural Relevance of an mHealth App for Hispanic/Latino Individuals Living With Rheumatoid Arthritis: Protocol for a Mixed Methods Study
Source: JMIR Res Protoc. 2026 Feb 13;15:e88401. doi: 10.2196/88401 (PMC12904348; doi:10.2196/88401)
Supplement: Peer Review Report 1 [file resprot-v15-e88401-s001.pdf]

January 21, 2025

Thais Alves, PhD
Department of Health Services & Research Administration
College of Public Health

Dear Dr. Alves

We are pleased to inform you that your application for the College of Public Health Innovation Fund has been recommended and approved for funding following a rigorous review process. The project " Evaluation of the Usability and Cultural Relevance of an mHealth App for Hispanics/Latinos with Rheumatoid Arthritis " has been awarded $15,000 for the funding period from February 1, 2025 to January 31, 2026.

In the appendix, we have included a summary statement of your pilot study. The reviewers have provided constructive feedback to enhance the study design and methodology, offering valuable insights to strengthen the proposed research. Their suggestions include refining data collection strategies, improving measurement tools, and addressing potential limitations. Additionally, they have recommended key areas to focus on for a future extramural grant application, such as expanding the study scope, incorporating longitudinal assessments, and strengthening collaborations with relevant stakeholders. We encourage you to carefully review their feedback and incorporate it into your project implementation and future funding pursuits.

As a recipient of the Innovation Fund, please ensure

- Use the funds within the approved timeframe and budget.
- Submit an extramural application within nine months of project completion.
- Provide 6-month and 12-month research progress reports.
- Identify and regularly meet with research mentors for guidance and feedback.
- Present project results at the PHIRE Conference.
- Acknowledge COPH as a sponsor in project-related publications and presentations.

Congratulations! We look forward to helping you reach your research goals and are excited to see your results.

Sincerely,

Dai

Daisy Dai, PhD
Associate Dean of Research

Appendix: Summary Statement

COLLEGE OF PUBLIC HEALTH
INNOVATION FUND 2024
SEED FUNDING FOR STRATEGIC PRIORITIES

Proposal Title	Evaluation of the Usability and Cultural Relevance of an mHealth App for Hispanics/Latinos with Rheumatoid Arthritis
PI Name	Thais Alves

REVIEWER #1

1. Significance

Strengths

- The project targets the significant health disparities experienced by Hispanic/Latino populations, particularly in rural areas, who suffer from rheumatoid arthritis (RA). The proposal cites recent reports and data supporting the prevalence of RA in diverse populations and the unique challenges faced by rural and Hispanic/Latino patients. If the aims are achieved, the project could provide new insights into how cultural relevance and usability in digital health interventions affect engagement and health management among Hispanic/Latino RA patients. This contributes to the broader understanding of culturally tailored healthcare technology. The findings can enhance the technical development of mobile health applications by integrating feedback mechanisms that account for cultural and linguistic diversity. Insights gained from the app usage patterns and user feedback may lead to refined app functionalities better suited to user needs. The successful completion of the aims may lead to improved strategies for patient-provider communication, especially for non-English speaking patients.

Weaknesses

- While the aims focus on understanding preferences and satisfaction, the evaluation of the actual clinical outcomes (e.g., improved disease management, reduced symptoms) may be limited in this phase. Recruiting a small number of patients may limit the generalizability of the findings especially given the varied cultural contexts within this population. Reliance of self-reported data. Questions remain about the long-term sustainability of the app and whether users will continue to engage with it beyond the study period. Will the participants have reliable internet connectivity?
- Another recommendation would be to provide a link to a video clip or snapshots or other visual aids on what the runRA app is, functionality, design, aesthetic appeal, etc; it was difficult to visualize

2. Investigator(s)

Strengths

- Dr. Alves, principal investigator, has expertise in mobile app evaluation, the application of qualitative methods, and experience with technology-based interventions with Hispanic/Latino populations. Dr. Cozad has expertise in digital health interventions (DHI) with a focus on evaluation and implementation. Dr. Horner will provide expertise in the management of activities, with specific responsibilities for manuscript formation and dissemination. Hayley Jurek, Research Recruitment and Outreach Coordinator, will provide expertise in patient recruitment through social media. This team has experience in the conduct of the proposed research project, successfully executing a project on a mHealth app for metastatic breast cancer that is supported by a P30 supplemental award from the National Cancer Institute. They appear to have complementary expertise and effective collaboration.

Weaknesses

- None

3. Innovation

Strengths

- Emphasizes a culturally-informed approach in digital health tools, recognizing the linguistic and cultural diversity of the Hispanic/Latino community.
- Utilizes an Iterative Convergent Mixed-methods design integrating qualitative and quantitative analyses, guided by the Framework for Reporting Adaptations and Modifications-Expanded (FRAME).
- Focusing on a digital health intervention aligns with contemporary healthcare trends that emphasize telehealth and mobile health resources as viable solutions to improve healthcare access—especially pertinent in rural areas.

Weaknesses

- What other apps are available, are there any similar ones and have they been successful, are there too many apps out there? How will it stand out? Will it be free?

4. Approach

Strengths

- The overall strategy, methodology, and analyses presented in the project proposal appear to be well-reasoned and appropriate for accomplishing the specific aims of the project. The investigators have effectively identified significant health disparities among Hispanic/Latino populations with rheumatoid arthritis (RA) and proposed a digital health intervention (RunRA) that could potentially address these issues. The proposal includes a comprehensive approach to assess health literacy, technology trust, and digital self-efficacy, as well as the cultural relevance and usability of the RunRA app. The mixed-methods design facilitates a thorough examination of both quantitative and qualitative data, allowing for a more nuanced understanding of the intervention's effectiveness.

Weaknesses

- The investigators acknowledge potential weaknesses in prior research and present strategies to mitigate these through cultural relevance and adaptability of the intervention. They have outlined how they will ensure a robust and unbiased approach, particularly through the use of established questionnaires and an iterative process for app improvement based on user feedback.

Moreover, the proposal addresses potential problems and alternative strategies, such as challenges in recruiting participants from minority groups. The plan to conduct individual interviews if focus groups cannot be arranged demonstrates flexibility and responsiveness to participant needs. The timeline for data collection and analysis is structured to establish feasibility and manage risks associated with the project's development.
Questions for investigators:
What are the specific benchmarks for success?
What happens if benchmarks are not met?

5. Environment

Strengths

Institutional Support: The proposal mentions the involvement of the University of Nebraska Medical Center (UNMC), which likely offers robust administrative support, access to research resources, and a commitment to health disparities research.
Access to Subject Populations: The project's focus on Hispanic/Latino populations living in rural Nebraska

is particularly relevant given the state's demographics. The investigators have access to a sizable population of individuals with rheumatoid arthritis who are likely to benefit from the RunRA app.
Collaborative Arrangements: The project benefits from collaboration among a multidisciplinary team with expertise in mobile health interventions, qualitative methods, and community outreach, enhancing the project's design and implementation.
Physical Resources and Equipment: The proposal mentions the Research, Education, Administration, and Development of Biomedical Informatics (READi) Core, which provides cutting-edge resources to support public health research. This access to technology and infrastructure is crucial for the successful execution of the project.
Unique Features of the Environment: The rural setting and the specific focus on health disparities among the Hispanic/Latino community provide unique insights and opportunities to tailor the intervention to address culturally relevant factors, which can enhance the likelihood of success.

Weaknesses

Rural populations may be geographically dispersed, making it difficult to reach potential participants for recruitment and data collection. Transportation issues can further hinder participation. The project targets a population that may already have low health literacy levels. Being in a rural environment may further compound this. This could complicate the understanding and effective use of the digital health intervention among participants. Internet access and smartphone usage may be less reliable in rural areas, potentially impacting the utilization of the RunRA app and the collection of data. Participants are limited to those who have access to a mobile device, which may not fully represent the views of the target population. Rural healthcare facilities may have fewer resources and personnel, which could limit the involvement of healthcare providers in the project or affect their willingness to engage with the app.

REVIEWER #2

The project addresses the important problem of health disparities (language barrier, access to care, lower health literacy) among Hispanics/Latinos (H/L) populations with rheumatoid arthritis (RA) in rural areas that contributes to higher RA disease activity and ultimately worse outcomes. The proposed digital health intervention, a Spanish-language version of the RunRA mHealth app, has the potential to improve patient-provider communication and disseminate culturally appropriate health-related information in a timely manner. The research team is highly qualified, although adding a rheumatologist or rheumatology clinician could further strengthen this dynamic research team by providing RA content expertise. While the study design is innovative, a clearer explanation of how successful completion of the aims could shift clinical practice (e.g., how could outcomes be improved) would make the proposal even stronger. Additionally, details about the RunRA mHealth app and its potential challenges and proposed solutions would provide a more robust approach. The institutional environment is supportive, although urban location constraints of the supporting institution may limit rural participation.

1. Significance

Strengths

- This proposal addresses an important problem of Hispanics/Latinos (H/L) with RA in rural areas experiencing higher RA disease activity owing to health disparities. Specifically, language barriers, access to care (transportation and provider shortage), lower health literacy that contribute to higher RA disease activity.
- Prior research is cited to support the importance of the problem being addressed.
- Solution being offered is a digital health intervention in the form of a Spanish language version of RunRA mHealth App tailored to H/L.

Weaknesses

- Prior research supporting the use of similar digital interventions to improve dissemination of health-related information or patient-provider communication in other populations with chronic conditions would improve the strength of this proposal.
- Existing research to support the usability and satisfaction of mHealth apps in other populations with chronic conditions would strengthen this proposal.

2. Investigator(s)

Strengths

- PI and members of the research team are well-situated to complete a successful project with experience in mobile app evaluation, qualitative research, technology-based interventions with H/L populations, evaluation and implementation of digital health interventions, and overall management of research activities including patient recruitment.

Weaknesses

- Expertise from a rheumatologist or a clinician who treats RA patients would strengthen this proposal by providing RA content expertise.

3. Innovation

Strengths

- New digital health technology is being investigated to determine its cultural relevance and usability in an underserved population with potential to improve patient-provider communication.
- Blending approaches of Iterative Convergent Mixed Methods study design and Implementation Science Framework (e.g., FRAME) to scale-up the intervention for improved delivery. Successful use has potential to demonstrate ability for future applications in other marginalized populations.

Weaknesses

- A description of how improving patient-provider communication could shift current clinical practice paradigms would strengthen this proposal.

4. Approach

Strengths

- Overall approach well-reasoned to accomplish specific aims. Potential weaknesses addressed (recruiting challenges, alternative to focus group with HCPs).
- Strategies to avoid personal bias and increase validity are discussed.

Weaknesses

- A thorough description of the RunRA mHealth app itself or similar apps used in other populations, potential problems and solutions, would strengthen this proposal.

5. Environment

Strengths

- The scientific environment is ideal to cultivate success of this project with institutional support from a major academic institution and an accompanying healthcare system that spans the state of NE.

Weaknesses

- Nebraska Medicine is primarily located in urban areas of the state which may limit rurality of participants.

REVIEWER #3

1. Significance

Strengths

- The project focuses on an unaddressed gap by exploring the use of culturally relevant digital health interventions (DHIs) for improving patient-provider communication for Hispanic/Latino (H/L) patients with rheumatoid arthritis (RA).
- It targets a vulnerable population with documented health disparities, emphasizing the compounded challenges faced by rural H/L communities in Nebraska.
- Recognizing the unique linguistic and cultural barriers faced by H/L populations ensures the intervention is patient-centered and tailored to the needs of the target group.

Weaknesses

- Potential Technological Barriers: The target population may face challenges in accessing or utilizing digital health interventions (e.g., limited internet access, low digital literacy, or lack of devices), which could hinder the intervention's adoption.
- Complexity of Cultural Tailoring: Developing a culturally and linguistically relevant intervention requires significant effort, expertise, and resources to ensure it adequately addresses the diverse needs within the H/L population.

2. Investigator(s)

Strengths

- Investigators have research expertise in digital health, mHealth app evaluation, qualitative research, digital health intervention, and comparative effectiveness.

Weaknesses

- None

3. Innovation

Strengths

- The use of an iterative convergent mixed methods design combined with the FRAME implementation science framework represents a novel approach for evaluating DHIs.
- By incorporating mobile applications to deliver culturally relevant and literacy-sensitive information, the study highlights the transformative potential of digital health technologies in reducing health disparities.
- The novelty of blending ICMM and FRAME for DHI evaluation establishes the project as a pioneering effort, contributing to methodological advancements in digital health research.

Weaknesses

- Combining ICMM & FRAME frameworks, while innovative adds layers of methodological complexity that may be difficult to execute effectively and require extensive expertise.

4. Approach

Strengths

- The integration of Iterative Convergent Mixed-methods and FRAME ensures a holistic approach to evaluating the intervention by combining qualitative and quantitative data.
- Including healthcare professionals and patients provides a dual perspective, enriching the understanding of the app's potential and challenges in patient-provider communication.
- The use of multiple recruitment methods, including social media and electronic medical record systems, imporoves the likelihood of reaching the target population while addressing potential barriers such as language and mistrust.
- The use of validated tools ensures the reliability and credibility of the study's outcomes.

- Leveraging resources from the READi Core and collaboration with bilingual professionals provides technical and cultural expertise, enhancing the study's quality and feasibility..

Weaknesses

- Despite multiple recruitment strategies, engaging a sufficient number of Hispanic/Latino participants and healthcare providers may be difficult.
- The timeline and scope of this study may restrict the number of iterations possible before finalizing the app for broader evaluation.

5. Environment

Strengths

- NA

Weaknesses

- NA

REVIEWER #4

1. Significance

Strengths

- The success of the proposed project would advance our understanding of how Hispanic/Latino rheumatoid arthritis (RA) patients' preferences and comfort in using digital health interventions, adapted to the patient's health literacy and interaction with the app, contribute to patient-doctor communication.

Weaknesses

- Given that our proposed research and objectives revolve around the RunRA application, a more comprehensive overview of the app's features and functionality is warranted.

2. Investigator(s)

Strengths

- The PI and collaborating researchers possess the requisite expertise in the proposed research endeavors, including patient recruitment and digital interventions in public health. Notably, the team has been actively engaged in designing the application, and they propose to adapt it for diverse linguistic and cultural contexts to cater to distinct user populations, which is feasible.

Weaknesses

- It is unclear whether the PI possesses the necessary technical background to spearhead the application revision independently and supervise additional technical personnel in the revision process.

3. Innovation

Strengths

- It is novel to have a culture-sensitive and language-adaptive app to help patients in managing symptoms and facilitating patient-doctor communication via m-Health app.

Weaknesses

- It is unclear what technology will be implemented or provided in the app, so more recent developments in generative AI, chatbot, etc might be useful for enhancing the innovation and novelty of the proposal and intervention.

4. Approach

Strengths

- The research design exhibited a rigorous evaluation plan, a well-structured recruitment strategy, and a robust sample, thereby ensuring the study's methodological soundness.

Weaknesses

- The proposal lacks technical details regarding the proposed mHealth app; it is unclear what functions will be provided to the patient, e.g., lack of description of the RunRA app.
- The plan to introduce the app to target patients, as well as the plan to encourage/control their interaction with the app, needs further clarification. Without proper design, it might end up lacking proper usage, which would impact the validity of the follow-up data collection. It is also unclear what app usage data will be collected for the analysis.
- It is unclear whether the Spanish version of the app is ready for use. If not, implementing the app may require some efforts, which could be one aim of the proposed work.

5. Environment

Strengths

- The institutional support and physical resources are adequate for the proposed work. The patient population and registry are also adequate for sampling and recruitment.

Weaknesses

- It would be helpful to consider an alternative plan if the selected participants lack proper equipment to install the app or have a stable internet connection to join the study.
